# Cystic Lymphangioma of the Chest Wall in a 5-Year-Old Male Patient: A Rare and Atypical Localization—A Case Report and Comprehensive Review of the Literature

**DOI:** 10.1155/2017/2083204

**Published:** 2017-10-23

**Authors:** Dimitrios Patoulias, Ioannis Patoulias, Christos Kaselas, Maria Kalogirou, Chatzopoulos Kyriakos, Farmakis Konstantinos, Thomas Feidantsis, Papacrivou Eleni

**Affiliations:** ^1^Department of Internal Medicine, General Hospital of Veria, Veria, Greece; ^2^1st Department of Pediatric Surgery, Aristotle University of Thessaloniki, General Hospital G. Gennimatas, Thessaloniki, Greece; ^3^General Hospital of Trikala, Trikala, Greece; ^4^Department of Pathology, General Hospital G. Gennimatas, Thessaloniki, Greece

## Abstract

Lymphangioma is a benign congenital malformation. The extremely rare and atypical localization of a lymphangioma in the chest wall was the real motive for the present case study. A 5-year-old boy was admitted to the Emergency Department of the 1st Department of Pediatric Surgery, Aristotle University of Thessaloniki, due to the presence of a mildly painful swelling in the left lateral chest wall, which was first noticed three months ago, after a blunt injury during sport. Physical examination revealed the presence of a palpable, spherical, painful, nut-sized subcutaneous lesion in the left lateral chest wall, respectively, with the anterior axillary line, at the height of the 6th to 7th intercostal space. Presence of ecchymosis on the overlying skin was also noticed. During palpation, we did not notice fluctuation, while transillumination was not feasible. Performance of ultrasonography, including Doppler color flow imaging, followed, depicting a subcutaneous cystic lesion, 2.1⁎3.2 cm in dimensions, without extension to the thoracic cavity. Scheduled surgical excision of the lesion was decided. Histopathological examination documented the diagnosis of cystic lymphangioma. Patient is still followed up on a 6-month basis. He remains asymptomatic, after 2 years, without indication of relapse.

## 1. Introduction

Lymphangioma is a benign congenital malformation characterized by proliferation of lymphatic vessels, resulting from the failure of communication between the primitive lymphatic sacs and the venous system, leading to the formation of a cystic structure [1]. Rarely, it is an acquired malformation, due to inflammation, injury, or fibrosis [2].

Based upon the histopathologic features, lymphangiomas are divided into three subtypes: the cystic (macrocystic) lymphangioma, which is the most common, the capillary (supermacrocystic) lymphangioma, and the corpus (cavernous, microcystic) lymphangioma, which is the most rare [3]. Macrocystic lymphangioma is classified as septated and nonseptated. As for etiology, according to embryologic mechanism, septated lymphangioma results from total obstruction of the primitive lymphatic sacs, while nonseptated lymphangioma from partial obstruction of them [4].

Their incidence in pediatric population rises up to 1 new case per 12000 births, while lymphangiomas represent almost 5-6% of all tumors in childhood [5]. Almost 95% of all lymphangiomas are detected in the cervical region or axillary cavity [6, 7]. Hancock et al. [8] reported that 31.4% of all lymphangiomas are found in the cervical region, 18.9% in the scalp and the face, 9.2% in the trunk, and 4.9% in the anterior chest wall and the axillary cavity. Chest wall localization of the lymphangiomas represents only 1% of all cases, with the majority of them located in the mediastinum [9, 10]. Omell et al. [11] were the first to describe a case of a chest wall lymphangioma in 1973. The extremely rare and atypical localization of a lymphangioma in the chest wall was the real motive for the present case study, after systematic and thorough research of the relevant literature [1, 3, 7, 9, 12, 13].

## 2. Case Report

**Figure 1:**
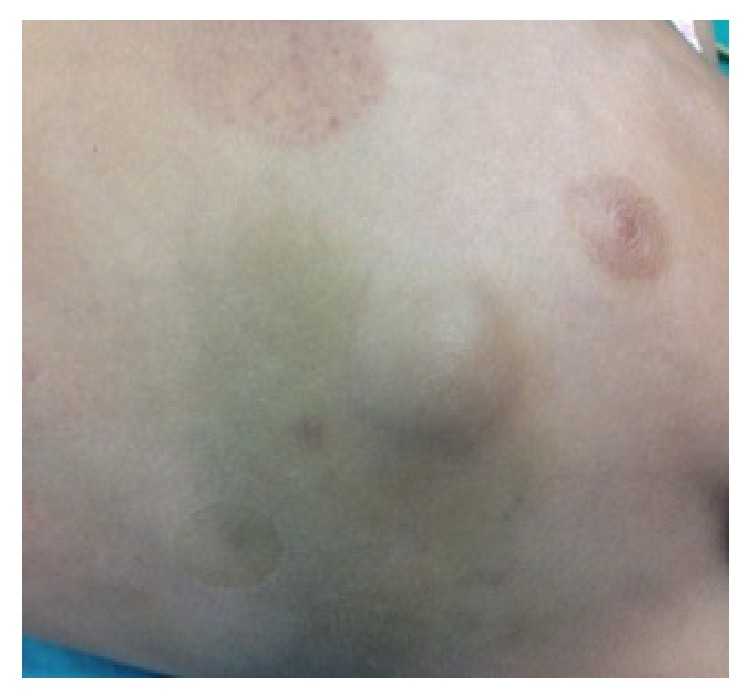
Presence of a palpable, spherical, painful, nut-sized subcutaneous lesion in the left lateral chest wall, respectively, with the anterior axillary line, at the height of the 6th to 7th intercostal space. Notice the ecchymosis on the overlying skin.

A 5-year-old male patient was admitted as an outpatient to the Emergency Department of the 1st Department of Pediatric Surgery, Aristotle University of Thessaloniki, due to the presence of a mildly painful swelling in the left lateral chest wall, which was first noticed about three months ago, after a blunt injury in the context of participation in sport. Physical examination revealed the presence of a palpable, spherical, painful, nut-sized subcutaneous lesion in the left lateral chest wall, respectively, with the anterior axillary line, at the height of the 6th to 7th intercostal space. Presence of ecchymosis on the overlying skin was also noticed (Figure 1).

Transillumination of the lesion was not feasible. No pathological signs were detected in face and left profile chest radiograph. Performance of ultrasonographic evaluation, including Doppler color flow imaging, followed, depicting a subcutaneous cystic lesion, 2.1*∗*3.2 cm in dimensions, without extension to the thoracic cavity. Solid insertion to the underlying epimysium was probable, according to the imaging findings. Lesion's periphery was lobulated, while no vascular structures, but thin septula (depicted as hyperechoic reflections), were found within it.

**Figure 2:**
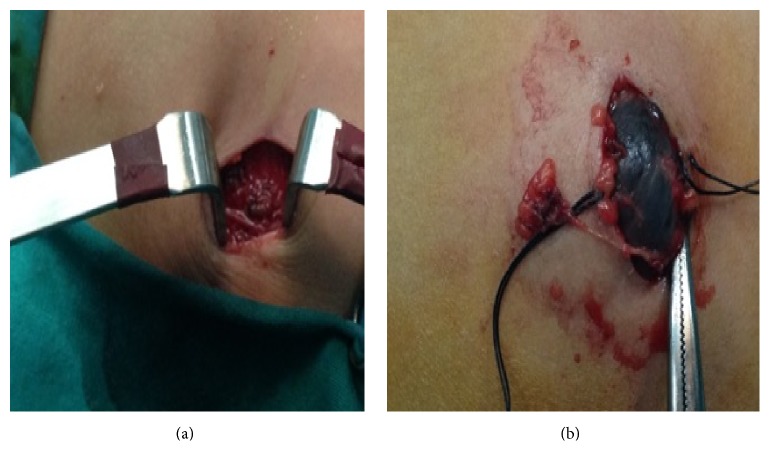
Procedure of lesion's excision.

Scheduled surgical excision of the lesion was decided, after conduction of the typical preoperative control, which was normal. With the patient under general endotracheal anesthesia, we conducted a cross section above the lesion, following the direction of Langer's lines. We then performed separation of the adhesions between the lesion and the adjacent subcutaneous fat tissue, but also of the adhesions between the posterior surface of the lesion and the underlying fascia of the anterolateral chest muscles (Figures 2(a) and 2(b)).

**Figure 3:**
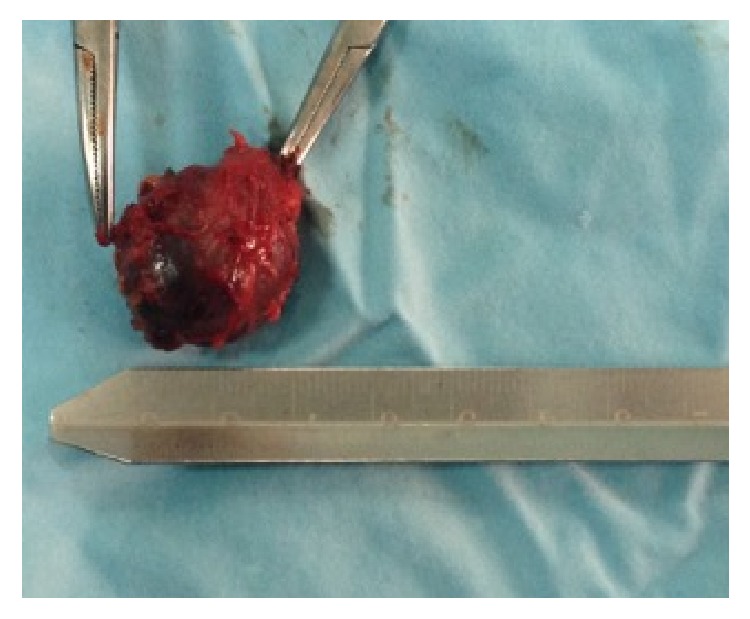
Total excised lesion, with a maximum diameter of 3.2 cm. Notice the intense hemorrhagic perfusion of its capsule.

The lesion was finally excised, without incision. We noticed the hemorrhagic perfusion of its capsule (Figure 3).

Hemostasis and repair of the surgical wound according to the anatomic order followed.

## 3. Results

**Figure 4:**
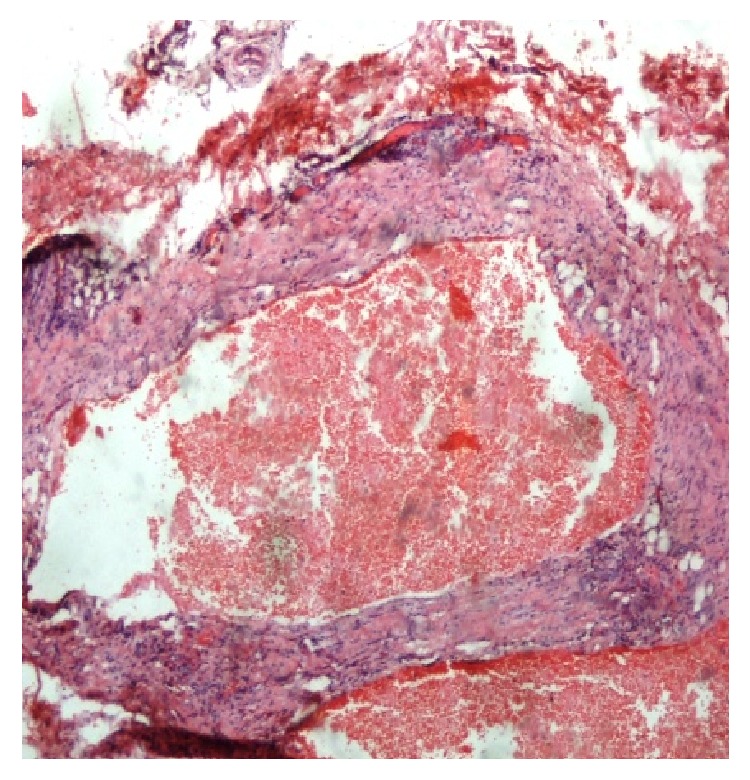
Notice the thickened fibrous capsule of the lymphangioma, invested by a flat endothelial layer, on the outer surface of which irregular striated muscle and elastic fibers are found. Thin septula are found within the lesion, while the subcavities are mainly occupied by hemorrhagic material. Notice also the presence of adipose tissue in the vicinity of the lesion (H/E 40x).

**Figure 5:**
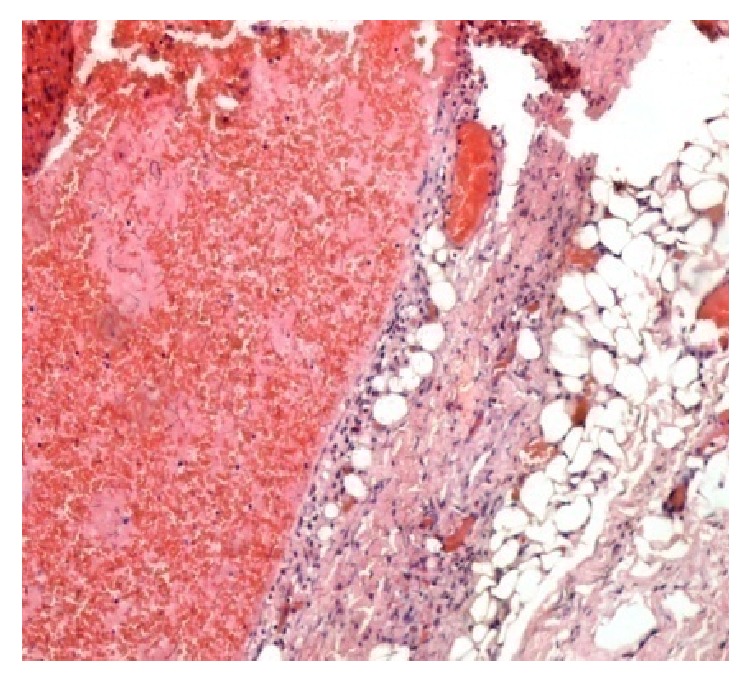
Higher magnification of Figure 4 (H/E, 100x).

Patient was easily awakened after the completion of the surgical procedure. Postoperative course was uneventful and he was discharged home on the 1st postoperative day in excellent general condition. Histopathological examination of the excised lesion documented the diagnosis of cystic lymphangioma. It highlighted the presence of a thickened fibrous capsule invested by a flat endothelial layer, on the outer surface of which irregular striated muscle and elastic fibers were found. Thin septula were found within the lesion, while the subcavities were mainly occupied by hemorrhagic material. Adipose tissue in the vicinity of the lesion was also found, corresponding to the subcutaneous fat tissue surrounding the lymphangioma (Figures 4 and 5).

Patient is still followed up on a 6-month basis. He remains asymptomatic, after 2 years, without indication of relapse.

## 4. Discussion

Goldstein et al. focus on the early prenatal diagnosis of lymphangiomas, between the 15th and 22nd week of gestation, in their study [14]. They consider that the early diagnosis of a chest wall cystic lymphangioma is not associated with poor prognosis, as in cases of lymphangiomas of other localization, due to chromosomal or anatomical anomalies, or even hydrops fetalis [14, 15]. Subsequently, based on the fact that prognosis is similar between a chest wall cystic lymphangioma and a delayed prenatally diagnosed cystic lymphangioma, it seems reasonable that development of a chest wall cystic lymphangioma does not result from obstruction of primitive lymphatic sacs, as their formation and conjunction with the venous system occur between 6th and 9th week of gestation [16–18].

Despite the presence of a cystic lymphangioma at birth in 50% of all cases and by the age of 2 years in 90% of all cases, diagnosis may be delayed significantly, as in our case [3, 19–22]. Mean age of diagnosis is 3 years of life. Rapid increase in size, inflammation, or intracystic bleeding, after injury, resulting in local pain, or pressure phenomena to adjacent anatomic structures, usually leads to diagnosis [22]. Intracystic bleeding can induce the formation of ecchymosis on the overlying skin, as in our case.

Physical examination and ultrasonography substantially contribute to the diagnostic approach of a chest wall cystic lymphangioma [1]. Transillumination, where possible, may also help the diagnosis, confirming the presence of a cystic lesion and excluding that of a solid structure [1]. In our case, there was no fluctuation of the lesion, while transillumination was not feasible, due to the increased tension and the intracystic bleeding.

Findings of ultrasonography and Doppler color flow are crucial for the preoperative evaluation, highlighting the cystic structure, the hypoechoic content, the lobulated periphery, the absence of vessels, and presence of thin septula [1]. In their study including 20 patients, Makni et al. [23] documented the diagnosis of cystic lymphangioma via ultrasonography in all of them. Evaluation of lymphangioma's dimensions and limits ruled out intrathoracic extension [20].

In cases of diagnostic doubt, conduction of CT or MRI may be required, in order to determine the possibility of intrathoracic extension of the lymphangioma and the affinity with the adjacent anatomic structures [9, 20, 24, 25]. In particular, CT may be crucial for the configuration of an operative plan, highlighting the affinity of the lymphangioma with the adjacent structures, mainly the large vessels. MRI offers even more detailed information regarding exertion of pressure to the airways and intrathoracic extension and viscus or bone involvement. In our case, ultrasonography revealed the presence of hyperechoic reflection within the lymphangioma, due to the intracystic bleeding. Essential information, as for the radical surgical excision of the lesion, is the confirmation or exclusion of invasive behavior of the lymphangioma [16, 26].

Surgical excision of a chest wall cystic lymphangioma is the treatment of choice [3]. Other suggested treatment options include radiation and injection of sclerosing agents like OKT 432 or ethanol 100% [3, 27]. Sasaki and Chiba applied successfully intrauterine treatment with OK-432 for a sizeable cystic lymphangioma diagnosed prenatally, while Ogita et al. describe two unsuccessful efforts for the intrauterine treatment of sizeable septated cystic lymphangiomas [28, 29].

Intralesional sclerotherapy (IS) constitutes an alternative treatment option in cystic lymphangiomas. Cahill et al. treated 17 patients with head and neck cystic lymphangiomas (10 macrocystic and 7 microcystic) by intralesional injection of doxycycline. The authors observed significant improvement in 11 out of 17 patients, while 8 out 11 featured a macrocystic type [30]. Impellizzeri et al. treated 8 patients with neck cystic lymphangiomas by CT guided IS using 98% sterile ethanol. In 7 out of those 8 patients cystic lymphangioma completely resolved after the first injection, while a second injection was required in the other patient [31]. Oliveira et al. [32], Russell et al. [33], and Mehmetoğlu [34] refer to conservative treatment of mesenteric lymphangiomas via IS, when (a) surgical excision is not feasible; (b) there is possibility of iatrogenic vascular injury during dissection; and (c) extended resection of the adjacent bowel may be required. In a retrospective study by Cheng [35], including 38 cases of head and neck lymphangiomas treated with IS using doxycycline, he evinced that 32 out of 38 patients (84.2%) were treated successfully, while desirable result was achieved after the first injection in 23 out of 32 patients (60.5%). He also documented that macrocystic lymphangiomas respond better to IS, compared to microcystic or mixed type.

We believe that complete excision is feasible and safe in cases of wall-localized cystic lymphangiomas, when performed by an experienced and skillful surgeon. In those cases, possibility of iatrogenic injury in the adjacent structures is minor. Besides, IS does not lack complications. According to previously mentioned study by Cahill et al., main observed complications were hemolytic anemia and delayed neurologic involvement [30].

However, when surgical intervention is not feasible, due to either the large dimensions or the increased likelihood of iatrogenic injury to adjacent anatomic structures, Turner et al. and Reinhardt et al. suggested either the conduction of systemic chemotherapy or the administration of interferon-a, but with poor results [36, 37].

Ozeki et al. administered propranolol at a single daily dose 2 mg/kg in 6 children with cystic lymphangioma, aged from 10 months to 19 years, over a period of 6 months [38]. As previously known, propranolol is a conservative treatment option in hemangiomas [39]. In two of those cases the researchers noticed a significant decrease in size of cystic lymphangiomas (30.6% and 22.9% resp.) within the 6 months of propranolol's administration. In one case they noticed moderate response to the therapeutic intervention (8% decrease in size), while in another case they noticed no alteration in lymphangioma's size, but recession of the symptoms. Finally, in two cases there was no response to administration of propranolol [38]. The researchers, except for the clinical evaluation of the patients, estimated also the serum levels of VEGF-A, VEGF-C, and VEGF-D (Vascular Endothelial Growth Factor) before the therapeutic intervention and 6 months after systematic administration of propranolol. Thus, they noticed that VEGF-A, VEGF-C, and VEGF-D levels were significantly reduced 6 months after administration of propranolol on a daily basis. Downregulation of the Rat mitogen activated protein kinase signaling pathway, leading to reduced expression of VEGF, may be the etiologic background, as for immunopathogenesis [38].

Histopathologic examination documents the diagnosis of a cystic lymphangioma, as in our case. The presence of striated muscle tissue adjacent to the posterior surface of the lymphangioma was indicative for its solid adhesion to the anterolateral chest muscles. Immunohistochemistry by using markers such as D_2_-40 and Lymphatic Vessel Endothelial Receptor-1 (LYVE-1) may be needed when lymphangioma should be differentially diagnosed from hemangioma or hemangioma-lymphangioma, by highlighting the presence of lymphatic endothelial cells on its inner surface, in cases of lymphangioma [40].

Since a cystic lymphangioma is fully excised, then there is practically no possibility of relapse [13, 41, 42]. Infiltration of the subject adipose tissue constitutes a negative prognostic factor, increasing the possibility of relapse after excision [25]. In cases of incomplete removal, relapse should be expected within the first 3 months postoperatively. Flanagan and Helwig report a case of relapse 7 years postoperatively, while Lee et al. report a case of a chest wall cystic lymphangioma that relapsed 19 years after surgical removal [3, 43]. Based on those mentioned above, we consider as substantial the long term periodic follow-up of our patient.
